# Auricular pressure as an adjuvant treatment for type 2 diabetes: data mining and meta-analysis

**DOI:** 10.3389/fendo.2024.1424304

**Published:** 2024-09-05

**Authors:** Yunfeng Yu, Qin Xiang, Xiu Liu, Yuman Yin, Siyang Bai, Rong Yu

**Affiliations:** ^1^ School of Traditional Chinese Medicine, Hunan University of Chinese Medicine, Changsha, Hunan, China; ^2^ Department of Endocrinology, The First Hospital of Hunan University of Chinese Medicine, Changsha, Hunan, China

**Keywords:** acupoints, auricular pressure, type 2 diabetes, meta-analysis, data mining, integrative medicine, traditional Chinese medicine

## Abstract

**Objective:**

This study aimed to explore the clinical efficacy and acupoint combinations of auricular pressure for treating type 2 diabetes.

**Methods:**

Eight common databases were searched for publications related to auricular pressure in type 2 diabetes as of November 2023. A meta-analysis was performed to assess the efficacy and safety of auricular pressure therapy. Data mining was used to analyze the core acupoints for auricular pressure.

**Results:**

Meta-analysis demonstrated that compared with the conventional treatment group, the combined auricular pressure and conventional treatment group had significantly reduced fasting blood glucose (mean difference [MD]: -0.93; 95% confidence interval [CI]: -1.17 to -0.68; *p* < 0.00001), 2-hour postprandial blood glucose (MD: -1.58; 95% CI: -2.04 to -1.12; *p* < 0.00001), glycated hemoglobin A1c (MD: -0.83; 95% CI: -1.19 to -0.48; *p* < 0.00001), total cholesterol (MD: -0.43; 95% CI: -0.72 to -0.14; *p* = 0.004), triglycerides (MD: -0.33; 95% CI: -0.64 to -0.03; *p* < 0.00001), systolic blood pressure (MD: -14.75; 95% CI: -24.46 to -5.05; *p* = 0.003), diastolic blood pressure (MD: -10.32; 95% CI: -20.14 to -0.50; *p* = 0.04), and body mass index (MD: -1.74; 95% CI: -2.61 to -0.87; *p* < 0.0001), while adverse events were comparable (RR: 0.84; 95% CI: 0.43 to 1.66; *p* = 0.61). Egger’s test revealed no publication bias (*p* = 0.715). Data mining identified AH_6a_, TF_4_, AT_4_, CO_18_, and CO_10_ as core acupoints for treating type 2 diabetes with auricular pressure.

**Conclusion:**

Auricular pressure safely improves blood glucose and lipid levels, blood pressure, and body mass index in patients with type 2 diabetes. A regimen consisting of AH_6a_, TF_4_, AT_4_, CO_18_, and CO_10_ is expected to serve as a complementary treatment for type 2 diabetes.

**Systematic review registration:**

www.crd.york.ac.uk/prospero/display_record.php?RecordID=524887, identifier CRD42024524887.

## 1 Introduction

Type 2 diabetes is a metabolic disease characterized by tissue-specific insulin resistance and pancreatic β-cell dysfunction (1). Epidemiological studies indicate that approximately 537 million people worldwide have diabetes, 90%–95% of whom have type 2 diabetes (2). Type 2 diabetes poses a significant threat to human health, leading to a series of complications such as diabetic nephropathy, retinopathy, cardiomyopathy, and peripheral neuropathy, which seriously jeopardize the physical and mental well-being of patients (3). The global health expenditure for diabetes and its complications was reported to be as high as $760 billion in 2019, placing a huge economic burden on patients, their families, and society (4). Although oral hypoglycemic drugs and insulin help in glycemic control (5), a small number of patients still experience poor glycemic control, and the potential adverse effects of these drugs remain a concern (6); as such, exploring safe, effective, and inexpensive adjuvant therapy for type 2 diabetes is important.

Acupuncture is a traditional Chinese medical technique with a long history of treating diseases by applying stimulation or pressure to specific acupoints. With greater interest in health management and natural therapies in recent years, the role of acupuncture in promoting health and treating diseases has received increasing attention (7, 8). Previous studies have shown that traditional acupuncture therapies, such as moxibustion, cupping, acupoint catgut embedding, acupoint application, and auricular pressure, help improve overall health outcomes (9–13). Auricular pressure is a treatment in which Wangbuliuxing seeds are applied to a patient’s auricular acupoints. It plays a role in treating diseases and strengthening the body through continuous acupoint stimulation (14). Auricular pressure is easy to perform, very safe, and well-tolerated (13); it is widely used in traditional Chinese medicine to treat various diseases such as allergic rhinitis, pain, and insomnia (13–15). Reports indicate that auricular pressure lowers blood glucose, reduces weight, and regulates lipid metabolism (16), indicating that it may be a potential supplementary therapy for type 2 diabetes. However, because of the lack of high-quality evidence, the specific benefits and risks of auricular pressure use in type 2 diabetes are still unclear. In addition, the acupoint selection scheme for auricular pressure treatment of type 2 diabetes has always been controversial. This study aimed to conduct a meta-analysis to assess the efficacy and safety of auricular pressure for type 2 diabetes and to explore a selection scheme of auricular acupoints through data mining.

## 2 Methods

This study strictly adhered to the Preferred Reporting Items for Systematic Reviews and Meta-Analyses (PRISMA) (17) and was registered in Prospero (CRD42024524887, www.crd.york.ac.uk/prospero/display_record.php?RecordID=524887).

### 2.1 Literature retrieval

A literature search was conducted by combining subject terms with extra terms. The subject terms included “auricular pressure” and “type 2 diabetes.”, and extra terms were obtained using MeSH and Sinomed. Relevant literature on the treatment of type 2 diabetes with auricular pressure published before November 2023 was queried in four English databases (Embase, PubMed, the Cochrane Library, and Web of Science) and four Chinese databases (China National Knowledge Infrastructure, China Science and Technology Journal Database, Wanfang, and Sinomed). No language restrictions were imposed.

### 2.2 Inclusion and exclusion criteria

Inclusion criteria: (і) Subjects meeting the 1999 World Health Organization diagnostic criteria for type 2 diabetes: Fasting blood glucose (FBG) ≥ 7.0 mmol/L or 2-hour postprandial blood glucose (2h-PBG) in oral glucose tolerance tests ≥ 11.1 mmol/L. (іі) The experimental group received conventional treatment combined with auricular pressure, while the control group received conventional treatment. Conventional treatment includes lowering blood glucose and blood pressure as well as regulating blood lipids, among others. (ііі) The efficacy endpoints included blood glucose (FBG, 2h-PBG, glycated hemoglobin A1c [HbA1c]), blood lipids (total cholesterol [TC], triglycerides [TG]), blood pressure (systolic blood pressure [SBP], diastolic blood pressure [DBP]), and body mass index (BMI). The safety endpoint was adverse events. (іv) The study design was a randomized controlled trial.

Exclusion criteria: (і) Duplicate data publication. (іі) Incomplete data. (ііі) Data not deemed usable.

### 2.3 Literature screening, data collection, and bias risk assessment

All literature was imported into the literature manager, and the included literature was selected based on the inclusion and exclusion criteria. The included literature was then classified, and basic information was extracted into a characteristics table. The risk of bias in the included studies was evaluated using the tools provided in Revman5.3. These tasks were independently completed by Qin Xiang and Xiu Liu, with any objections ruled upon by Yu.

### 2.4 Statistical analysis

Meta-analysis was performed using Revman5.3. The mean difference (MD) and risk ratio (RR) were used as effect sizes for continuous and dichotomous variables, respectively. Heterogeneity was assessed using the I^2^ test; when I^2^ < 50%, a fixed-effect model was used for analysis, while when I^2^ ≥ 50%, a random-effect model was used. A leave-one-out sensitivity analysis was used to assess the robustness of the results. Egger’s test was performed using Stata15.0 to evaluate publication bias, with *p* > 0.1 indicating no publication bias. Frequency analysis was performed to obtain common acupoints for auricular pressure treating type 2 diabetes using Traditional Chinese Medicine Case Cloud V2.3, with a frequency threshold of ≥ 30%. The common acupoints were imported into SPSS Modeler 18.0 for association rule analysis, and the following parameters were set to obtain the core auricular acupoint combinations for type 2 diabetes: Apriori model, support ≥ 50%, confidence ≥ 80%, and lift ≥ 1.0. The acupoints that comprised these core acupoint combinations were defined as core acupoints.

## 3 Results

### 3.1 Literature screening

**Figure 1:**
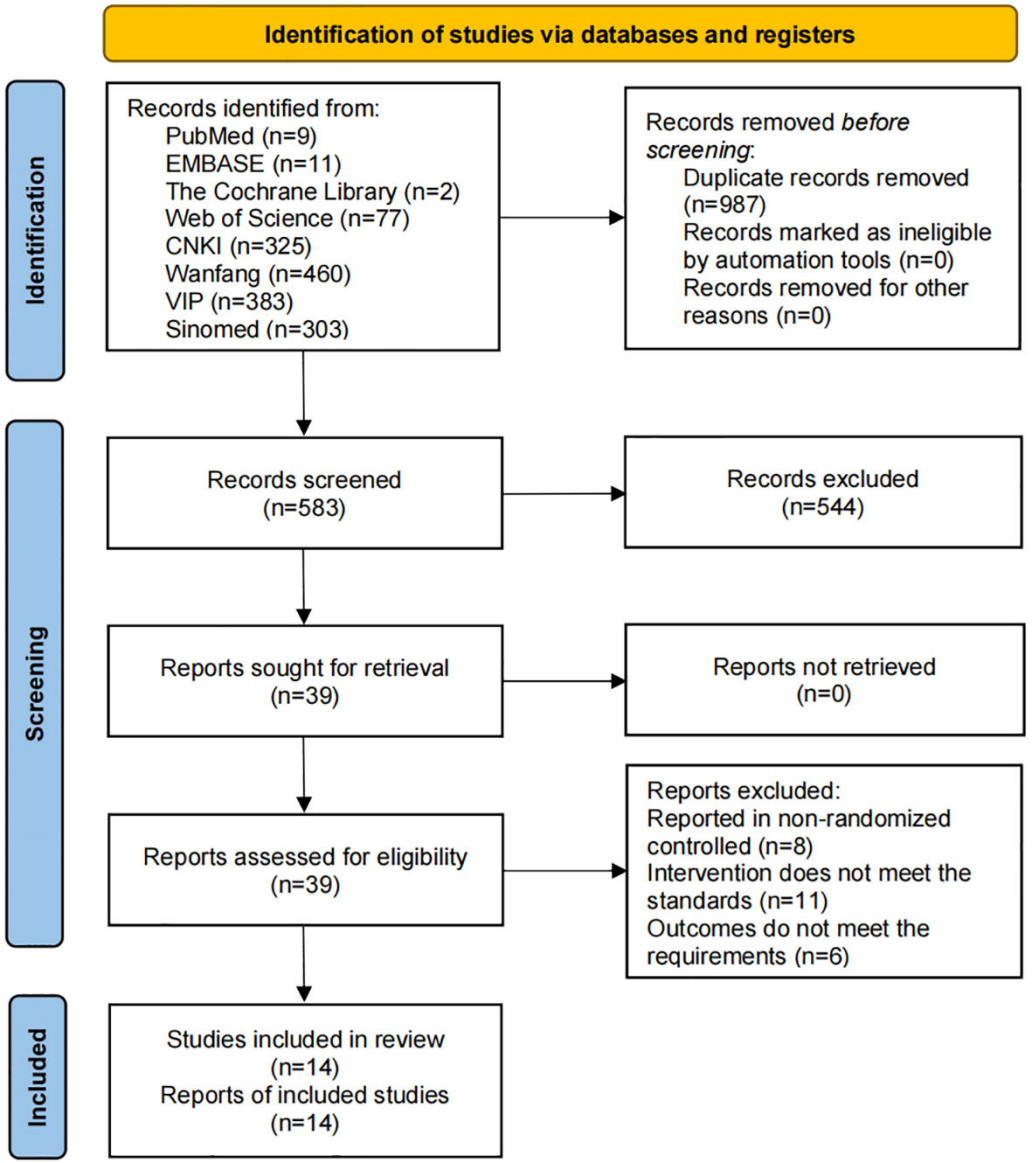
Flowchart of literature screening.

The database contained 1570 relevant studies. Ultimately, 14 articles were included (18–31) after 987 were excluded because of duplication, and 569 were excluded because they did not meet the inclusion criteria (**Figure 1**).

### 3.2 Basic characteristics of included studies

Fourteen clinical trials were included (18–31) with a total sample size of 1221 cases, all conducted in China with publication years ranging from 2014 to 2022. A total of 612 cases received auricular pressure in combination with conventional treatment, while 609 received conventional treatment alone (**Table 1**).

**Table 1:** Basic characteristic of the included studies.

Study	Sample (E/C)	Male (%)	Age (years)	Disease duration (years)	Intervention frequency	Treatment duration (days)	Acupoints
Gao Q (19)	28/28	55.4	58.3	8.6	once every two days	28	AT_4_, AH_6a_, TF_4_, CO_18_
Hu DX (20)	100/100	60.0	45.0	/	once every three days	30	CO_18_, CO_10_, CO_15_, CO_12_, CO_17_, AT_4_
Huang W (21)	40/38	39.7	68.6	10.0	twice a week	84	CO_18_, CO_11_, TF_4_, TG_2_
Jian YM (22)	50/50	54.0	61.2	4.5	once every three days	54	CO_11_, AH_6a_, AT_4_, CO_18_, CO_12_, CO_14_, CO_13_, CO_4_, CO_10_, CO_17_, CO_6_, CO_7_, TF_4_
Luo LQ (23)	50/50	54.0	52.9	/	once every two days	30	AT_4_, AH_6a_, TF_4_, CO_18_
Ma XM (24)	30/30	50.0	54.0	6.1	twice a week	21	CO_11_, CO_18_, CO_17_, CO_4_, CO_10_
Shen ZQ (25)	50/50	50.0	57.8	7.2	once a week	56	AT_4_, TF_4_, AH_6a_, CO_15_, CO_13_, CO_10_
Wang P (26)	50/50	50.0	58.4	5.3	once every five days	30	AH_6a_, AT_4_, CO_18_, TF_4_, CO_12_, CO_10_, CO_15_, TG_2_, CO_7_
Wang XJ (27)	31/31	61.3	54.8	8.2	once every three days	84	CO_11_, CO_18_, CO_13_, CO_10_, CO_9_
Wang XM (28)	52/50	53.9	48.7	/	once a week	84	CO_18_, TG_2p_, CO_14_, TG_1_, CO_13_, CO_4_, CO_9_
Xiao Q (29)	22/23	51.1	57.8	/	once every two days	28	AT_4_, AH_6a_, TF_4_, CO_18_
Xu YW (30)	30/30	61.7	59.1	6.7	once every five days	14	P_S_, CO_12_, CO_10_, TF_4_, AH_6a_, AT_4_, CO_18_
Yang YY (31)	30/30	41.7	58.2	/	once every two days	14	TF_4_, CO_15_, AT_4_, AH_6a_, CO_18_, CO_13_, CO_14_, CO_4_, CO_10_
Zheng YP (18)	49/49	26.5	58.7	6.9	once every five days	180	TF_4_, AT_3_, AT_1_, AT_4_, LO_4_, SMSC, CO_4_, CO_12_, CO_17_, CO_18_, CO_14_, CO_13_, CO_11_, CO_15_, CO_10_

SMSC, ear acupoint named Shuimian Shenchen.

### 3.3 Bias risk assessment

**Figure 2:**
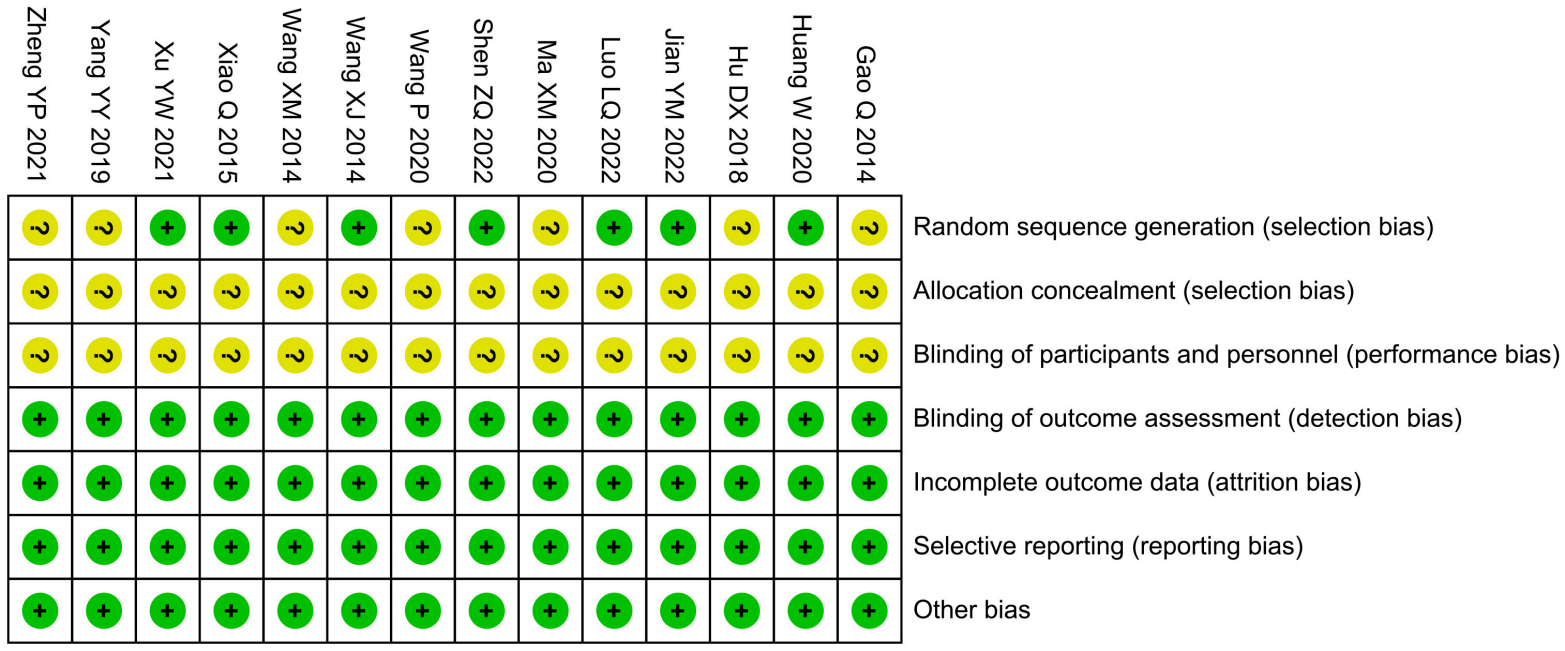
Risk of bias summary.

The bias risk of randomization in seven studies was unclear, while that of allocation concealment, as well as blinding of participants and personnel, was unclear in 14 studies. The risk of bias in other areas was low (**Figure 2**).

### 3.4 Efficacy endpoint

#### 3.4.1 Blood glucose

**Figure 3:**
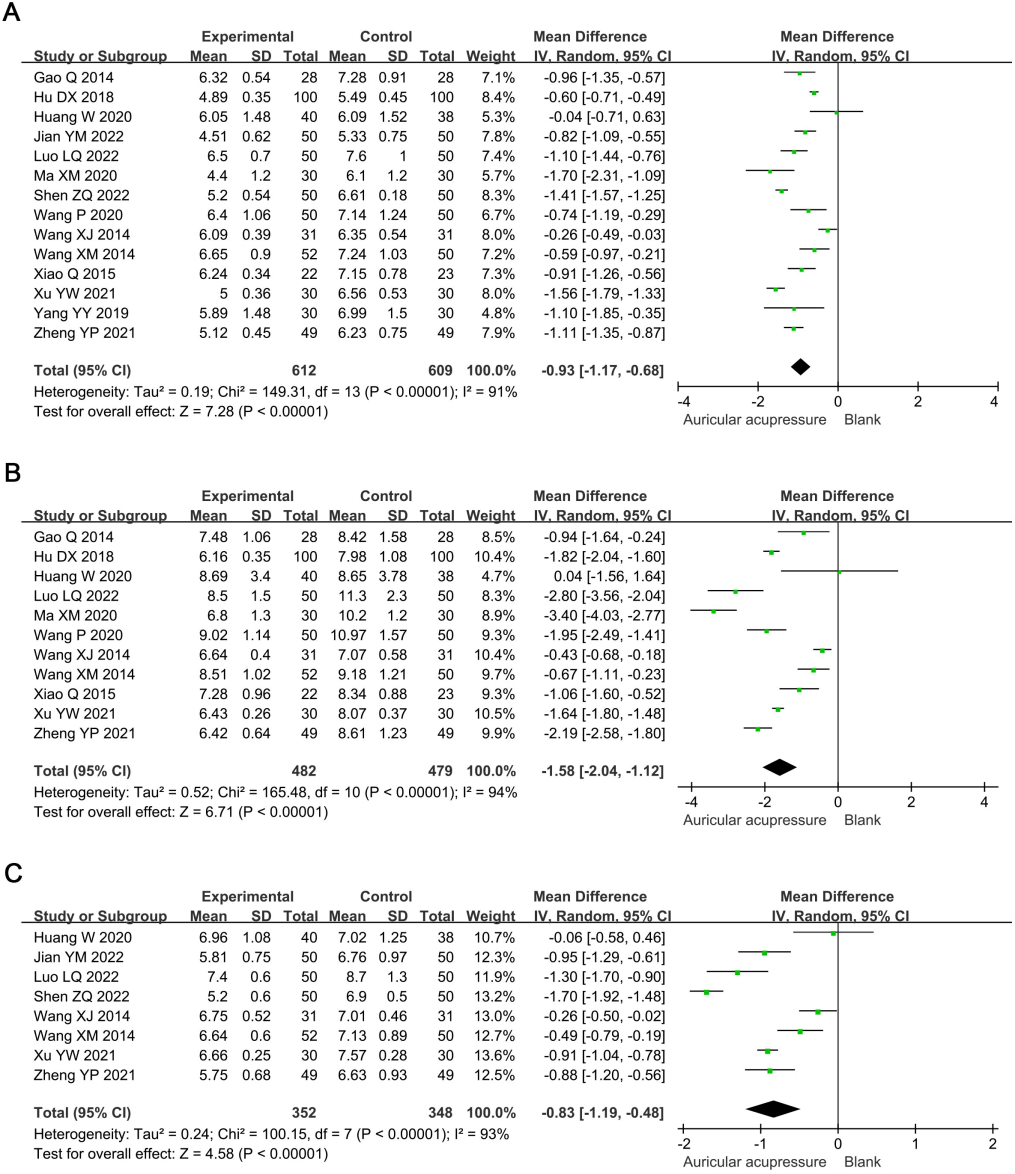
Meta-analysis results of blood glucose in T2D treated with auricular pressure. **(A)** FBG; **(B)** 2h-PBG; **(C)** HbA1c. FBG, fasting blood glucose; PBG, postprandial blood glucose; HbA1c, glycosylated hemoglobin A1c.

Meta-analysis showed that combined treatment significantly reduced FBG by 0.93 mmol/L (MD: -0.93, 95% confidence interval [CI]: -1.17 to -0.68; *p* < 0.00001), 2h-PBG by 1.58 mmol/L (MD: -1.58, 95% CI: -2.04 to -1.12; *p* < 0.00001), and HbA1c by 0.83% (MD: -0.83, 95% CI: -1.19 to -0.48: *p* < 0.00001) compared with the conventional treatment group (**Figure 3**). Sensitivity analysis showed that the results were robust.

#### 3.4.2 Blood lipids

**Figure 4:**
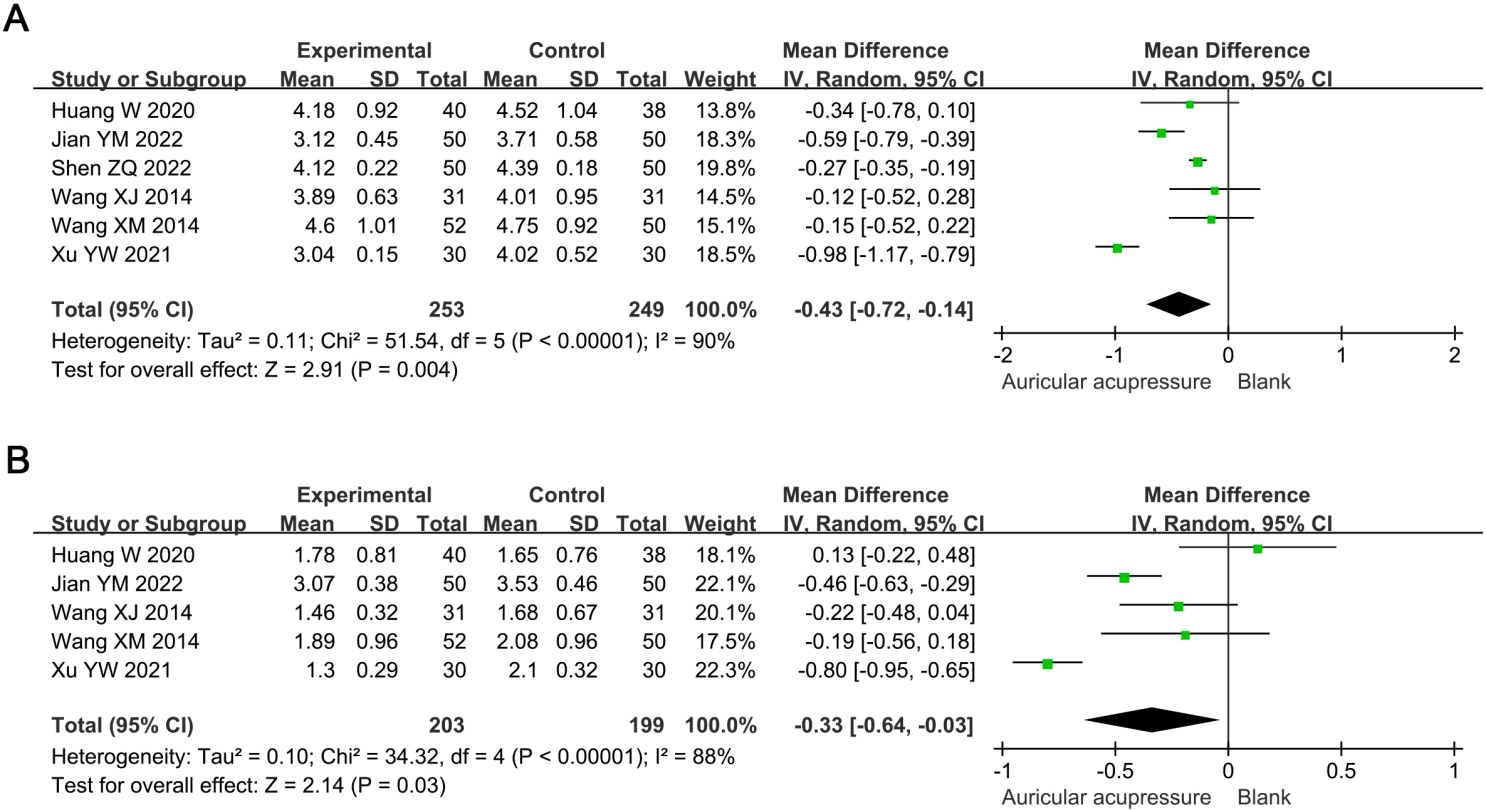
Meta-analysis results of blood lipids in T2D treated with auricular pressure. **(A)** TC; **(B)** TG. TC, total cholesterol; TG, triglyceride.

Compared with the conventional treatment group, the combined treatment significantly reduced TC by 0.43 mmol/L (MD: -0.43, 95% CI: -0.72 to -0.14; *p* = 0.004) and TG by 0.33 mmol/L (MD: -0.33, 95% CI: -0.64 to -0.03; *p* = 0.03) (**Figure 4**). Sensitivity analysis showed that the results for TC were robust, whereas those for TG were not; the significance of TG changes disappeared after excluding Jian YM 2022 (MD: -0.29, 95% CI: -0.74 to 0.16; *p* = 0.21) or Xu YW 2021 (MD: -0.21, 95% CI: -0.47 to 0.04; *p* = 0.09).

#### 3.4.3 Blood pressure

**Figure 5:**
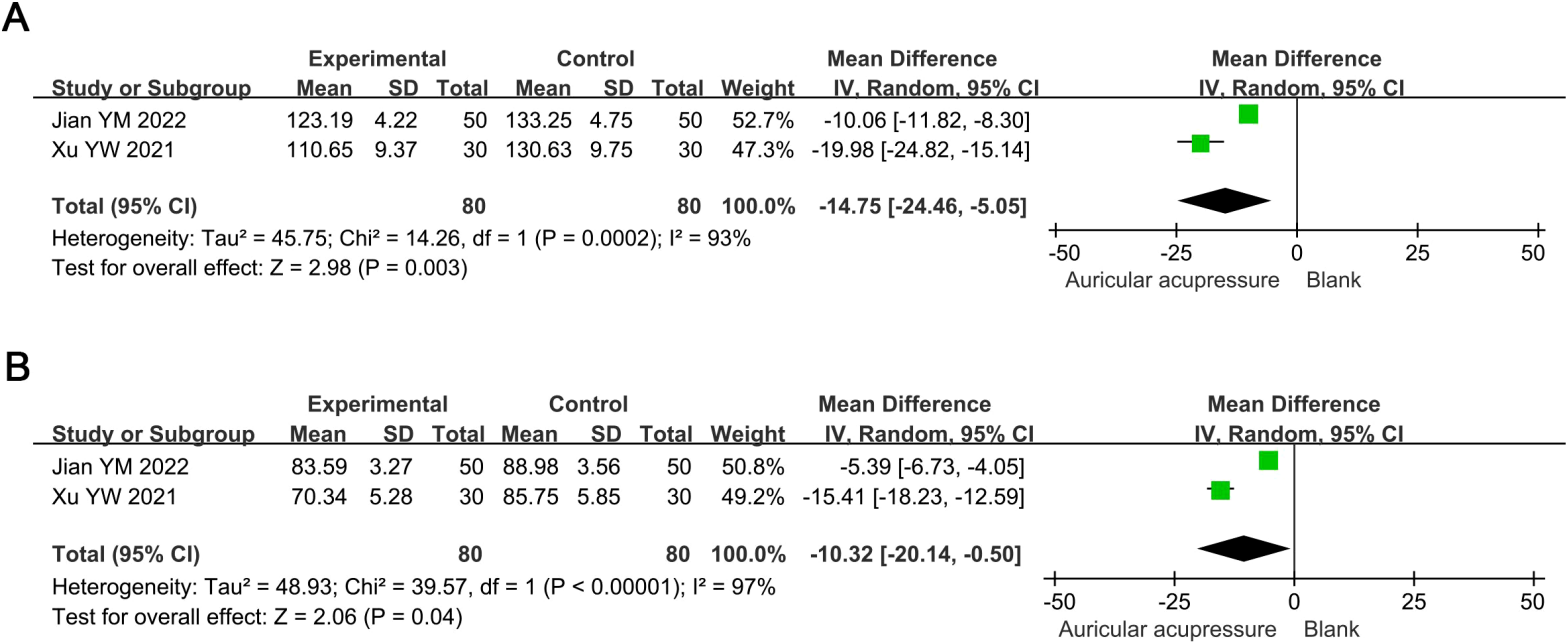
Meta-analysis results of blood pressure in T2D treated with auricular pressure. **(A)** SBP; **(B)** DBP. SBP, systolic blood pressure; DBP, diastolic blood pressure.

The combined treatment significantly reduced SBP by 14.75 mmHg (MD: -14.75, 95% CI: -24.46 to -5.05; *p* = 0.003) and DBP by 10.32 mmHg (MD: -10.32, 95% CI: -20.14 to -0.50; *p* = 0.04) compared to the conventional treatment group (**Figure 5**). Sensitivity analysis indicated these results were robust.

#### 3.4.4 BMI

**Figure 6:**
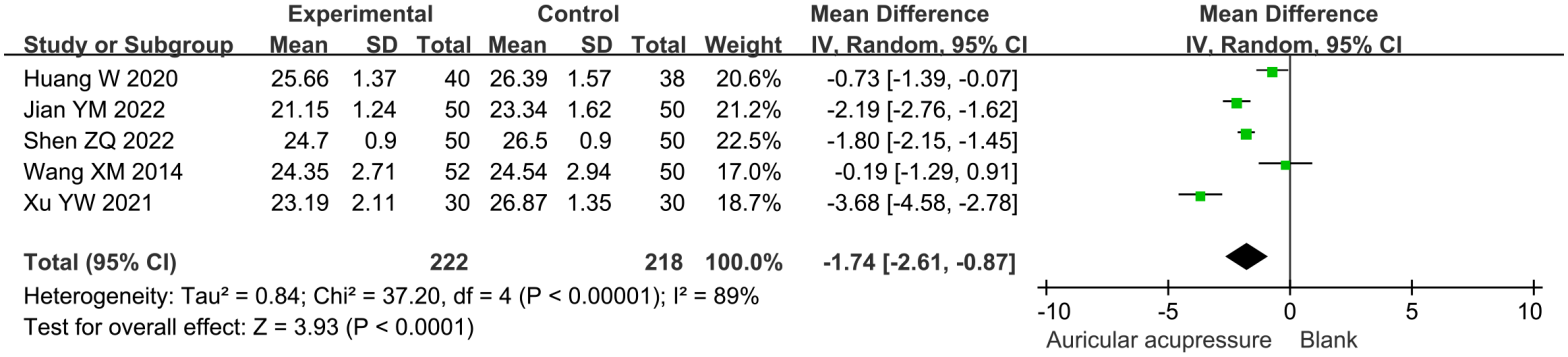
Meta-analysis results of BMI in T2D treated with auricular pressure. BMI, body mass index.

The combined treatment significantly decreased BMI by 1.74 kg/m^2^ (MD: -1.74, 95% CI: -2.61 to -0.87; *p* < 0.0001) compared with the conventional treatment group (**Figure 6**). Sensitivity analysis demonstrated the robustness of this result.

### 3.5 Safety endpoints

**Figure 7:**
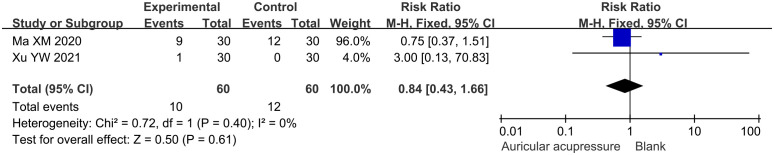
Meta-analysis results of adverse events in T2D treated with auricular pressure.

The incidence of adverse events in the combined treatment group was 16.7%, while that in the conventional treatment group was 20.0%. The adverse events in both groups were comparable (RR: 0.84, 95% CI: 0.43 to 1.66; *p* = 0.61) (**Figure 7**). Sensitivity analysis confirmed the robustness of this result.

### 3.6 Subgroup analysis

Subgroup analysis with FBG as the outcome indicator was used to investigate the impact of factors such as treatment frequency, treatment duration, and number of acupoints on the efficacy of auricular pressure treatment for type 2 diabetes. Frequencies of ≤ 3 days (MD: -0.77, 95% CI: -0.99 to -0.55; *p* < 0.00001) and > 3 days per session (MD: -1.07, 95% CI: -1.39 to -0.75; *p* = 0.007) significantly reduced FBG. Auricular pressure treatment durations of ≤ 30 (MD: -1.07, 95% CI: -1.41 to -0.72; *p* = 0.0004) and > 30 days (MD: -0.74, 95% CI: -1.18 to -0.31; *p* = 0.0008) significantly lowered FBG. Five or less (MD: -0.82, 95% CI: -1.25 to -0.40; *p* = 0.0001) and > 5 acupoints (MD: -1.00, 95% CI: -1.32 to -0.67; *p* < 0.00001) of auricular pressure significantly reduced FBG (**Table 2**).

**Table 2:** Subgroup analysis results of auricular pressure for T2D.

Subject	Subgroup	I^2^	MD (95% CI)	*p* value
Treatment frequency	Reach or exceed once every 3 days	77	-0.77 (-0.99, -0.55)	<0.00001
	Less than once every 3 days	86	-1.07 (-1.39, -0.75)	0.007
Treatment duration	≤ 30 days	90	-1.07 (-1.41, -0.72)	0.0004
	> 30 days	94	-0.74 (-1.18, -0.31)	0.0008
Number of acupoints	≤ 5 acupoints	86	-0.82 (-1.25, -0.40)	0.0001
	> 5 acupoints	93	-1.00 (-1.32, -0.67)	<0.00001

### 3.7 Publication bias

**Figure 8:**
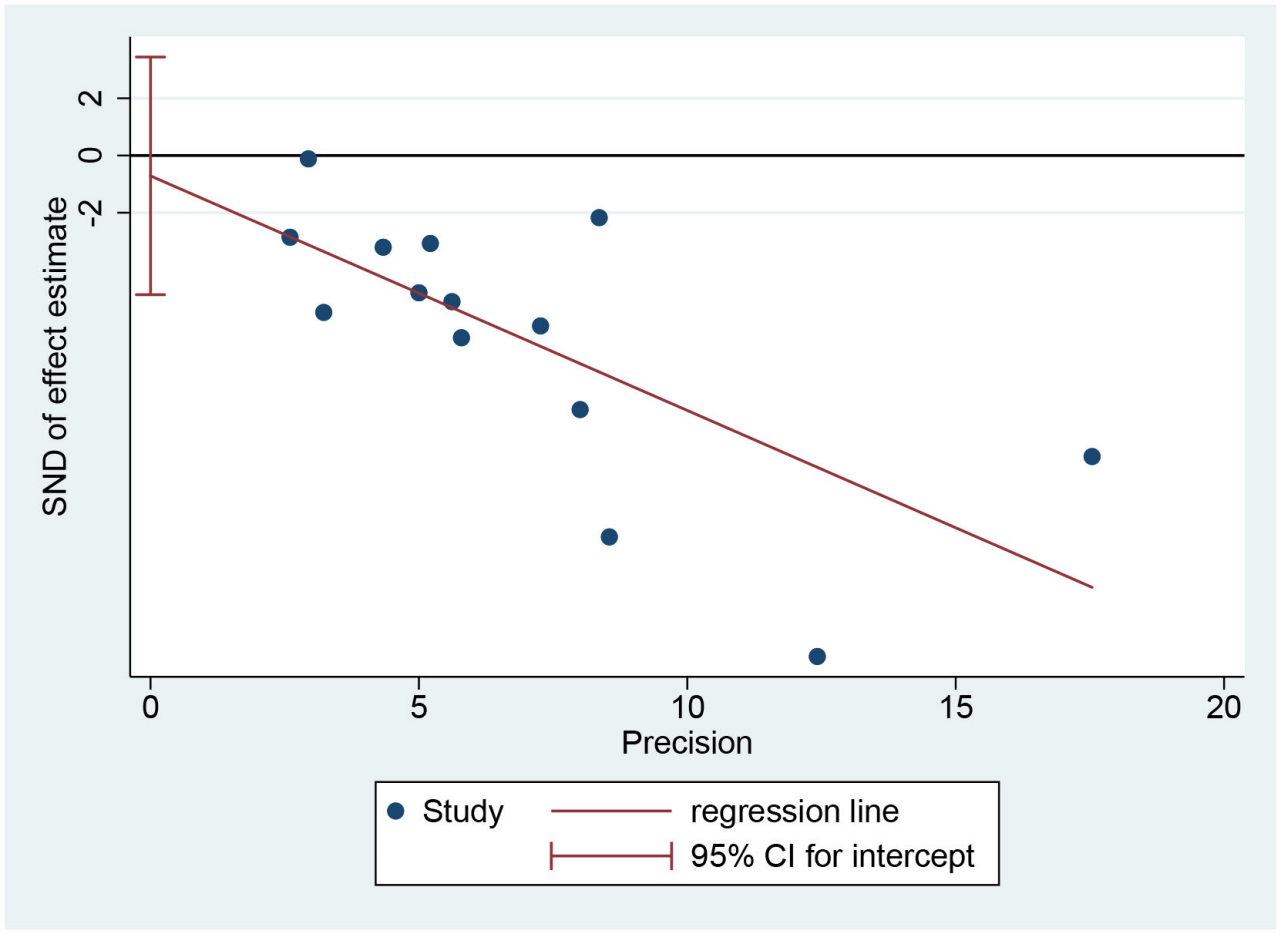
Publication bias based on Egger test.

When FBG level was defined as the primary efficacy endpoint, Egger’s test showed a *p*-value of 0.715, indicating no significant publication bias (**Figure 8**).

### 3.8 Frequency analysis

Fourteen auricular pressure protocols were included in the frequency analysis, with a total frequency of 98. The protocol involved 23 auricular acupoints (**Table 3**). The frequencies of CO_18_ (92.9%), AT_4_ (71.4%), TF_4_ (71.4%), CO_10_ (64.3%), AH_6a_ (57.1%), CO_13_ (42.9%), CO_11_ (35.7%), CO_12_ (35.7%), CO_15_ (35.7%), and CO_4_ (35.7%) were all above 30.0%, making them common acupoints in the treatment of type 2 diabetes using auricular pressure.

**Table 3:** Frequency analysis results of auricular pressure for T2D.

Rank	Acupoint	Frequency (n/%)	Location
1	CO_18_	13 (92.9%)	Inside the intertragic notch, at the bottom of the cavum concha.
2	AT_4_	10 (71.4%)	On the medial side of the antitragus.
3	TF_4_	10 (71.4%)	At the upper 1/3 of the posterior part of the triangular fossa.
4	CO_10_	9 (64.3%)	Inferior to the posterior part of the inferior antihelix crus.
5	AH_6a_	8 (57.1%)	At the junction of the front end of the inferior antihelix crus and the inner edge of the helix.
6	CO_13_	6 (42.9%)	Below the BD line, at the upper back part of the cavum concha.
7	CO_11_	5 (35.7%)	At the upper back part of the cymba concha.
8	CO_12_	5 (35.7%)	At the lower back part of the cymba concha.
9	CO_15_	5 (35.7%)	At the central depression of the cavum concha.
10	CO_4_	5 (35.7%)	At the end point of the helix crus.
11	CO_14_	4 (28.6%)	Around the CO_15_ and CO_16_.
12	CO_17_	4 (28.6%)	Posterior and inferior to the orifice of the external auditory meatus, between the CO_14_ and CO_18_.
13	CO_7_	2 (14.3%)	At the front 1/3 between the AB line and the helix crus with part of the helix.
14	CO_9_	2 (14.3%)	At the middle part inferior to the inferior antihelix crus.
15	TG_2_	2 (14.3%)	At the lower 1/2 on the outer side of the tragus.
16	AT_1_	1 (7.1%)	At the front part of the outer side of the antitragus.
17	AT_3_	1 (7.1%)	At the rear part of the outer side of the antitragus.
18	CO_6_	1 (7.1%)	At the middle 1/3 between the AB line and the helix crus with part of the helix.
19	LO_4_	1 (7.1%)	At the anterior middle of the front of the lobe.
20	TG_2p_	1 (7.1%)	At the lower tip of the free edge of the tragus.
21	TG_1_	1 (7.1%)	At the upper 1/2 on the outer side of the helix.
22	P_S_	1 (7.1%)	At the groove of the antihelix as well as the groove of the superior and inferior antihelix crus.
23	SMSC	1 (7.1%)	At the back of the auricle, corresponding to the point for neurasthenia.

SMSC, ear acupoint named Shuimian Shenchen.

### 3.9 Association rule analysis

**Figure 9:**
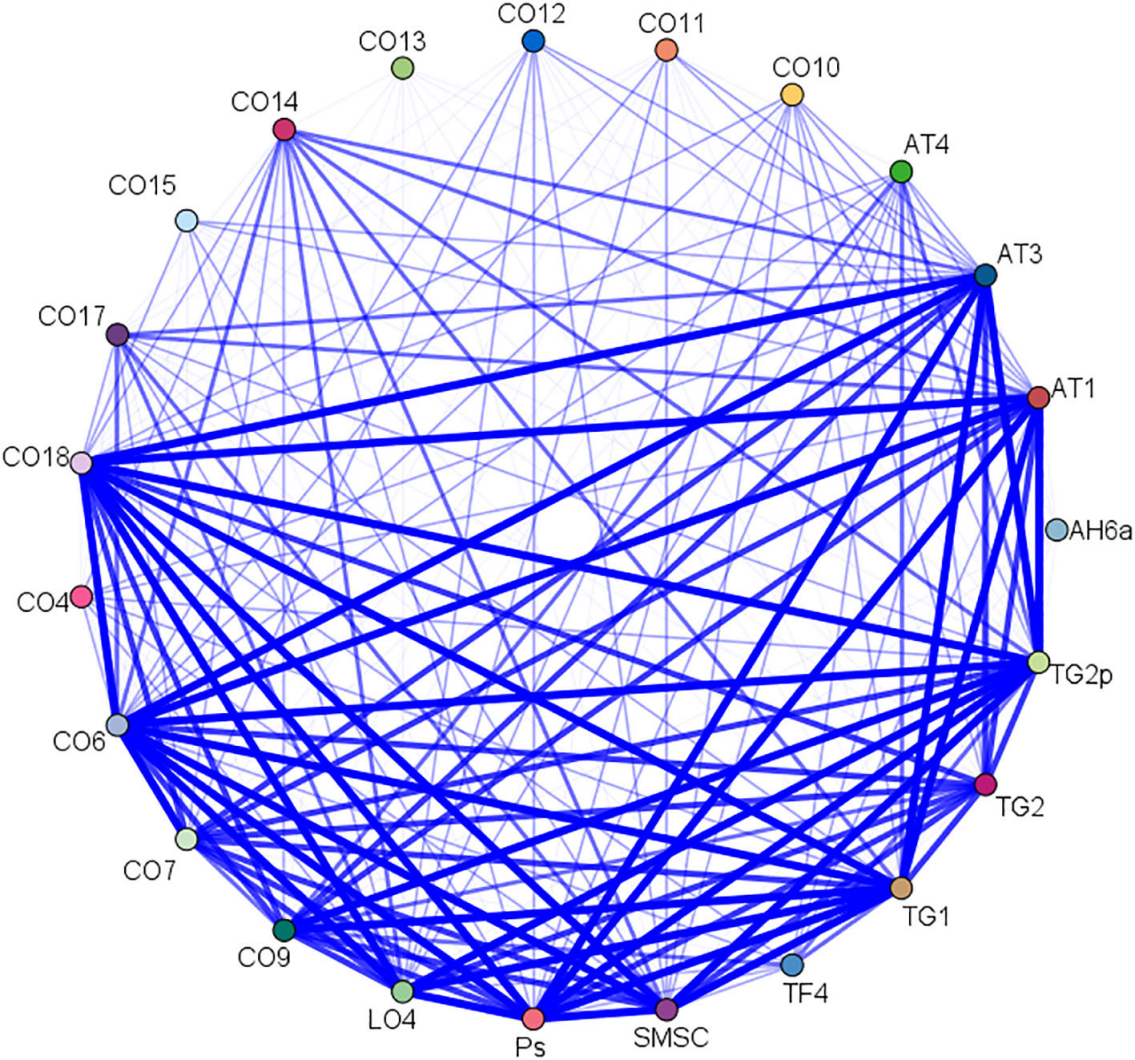
Network relationship of acupoints.

A total of 21 acupoint combinations were obtained through association rule analysis (**Table 4**). The AT_4_-TF_4_ combination had the highest support, confidence, and lift values, while the AH_6a_-TF_4_-AT_4_-CO_18_ combination had the largest number of acupoints. These core acupoint combinations were composed of acupoints AH_6a_, TF_4_, AT_4_, CO_18_, and CO_10_. The network relationships are shown in **Figure 9**.

**Table 4:** Association rule analysis results of auricular pressure for T2D.

Preceding paragraph	Behind paragraph	Support/%	Confidence/%	Benefits
AT_4_	TF_4_	71.4	90.0	1.3
TF_4_	AT_4_	71.4	90.0	1.3
CO_18_	TF_4_	71.4	90.0	1.0
CO_18_	AT_4_	71.4	90.0	1.0
CO_18_	CO_10_	71.4	90.0	1.0
AH_6a_	TF_4_	71.4	80.0	1.4
AH_6a_	AT_4_	71.4	80.0	1.4
AH_6a_	TF_4_, AT_4_	64.3	88.9	1.6
AT_4_	TF_4_, CO_18_	64.3	88.9	1.2
TF_4_	AT_4_, CO_18_	64.3	88.9	1.2
CO_18_	TF_4_, AT_4_	64.3	88.9	1.0
TF_4_	AH_6a_	57.1	100.0	1.4
AT_4_	AH_6a_	57.1	100.0	1.4
AT_4_	AH_6a_, TF_4_	57.1	100.0	1.4
TF_4_	AH_6a_, AT_4_	57.1	100.0	1.4
AH_6a_	TF_4_, AT_4_, CO_18_	57.1	87.5	1.5
TF_4_	AH_6a_, CO_18_	50.0	100.0	1.4
AT_4_	AH_6a_, CO_18_	50.0	100.0	1.4
AT_4_	AH_6a_, TF_4_, CO_18_	50.0	100.0	1.4
TF_4_	AH_6a,_ AT_4_, CO_18_	50.0	100.0	1.4
TF_4_	AT_4_, CO_10_	50.0	85.7	1.2

## 4 Discussion

### 4.1 Background and significance

Auricular pressure is a treatment that applies Wangbuliuxing seeds to the auricle acupoints under the guidance of traditional Chinese medicine theory (32). Owing to its simplicity and high safety, it has been utilized by traditional Chinese medicine practitioners to treat various conditions such as insomnia, pain, and allergic rhinitis (13–15). In 1994, Chen H and colleagues (33) first reported the application of auricular pressure in patients with diabetes in a clinical trial, finding that auricular pressure promoted insulin secretion and lowered blood glucose in patients with diabetes. Since then, many studies have suggested that auricular pressure may be a potential adjunct treatment for type 2 diabetes. However, because of the lack of high-quality evidence, the benefits and risks of auricular pressure use in type 2 diabetes remain uncertain. To the best of our knowledge, this is the first meta-analysis and data mining study examining auricular pressure for type 2 diabetes, aiming to provide evidence-based support for its clinical application.

### 4.2 Efficacy evaluation

Meta-analysis demonstrated that auricular pressure combined with conventional treatment significantly reduced FBG by 0.93 mmol/L, 2h-PBG by 1.58 mmol/L, and HbA1c by 0.83% compared with conventional treatment alone. FBG is an indicator used to diagnose and monitor diabetes, reflecting the basal insulin secretion function of pancreatic β-cells. 2h-PBG is an important indicator that reflects postprandial insulin secretion and predicts changes in renal function. HbA1c is an important indicator of blood glucose status and cardiovascular risk over the past 90 days. The benefits of auricular pressure on FBG, 2h-PBG, and HbA1c levels indicate that it regulates blood glucose levels and improves prognosis. In addition, Shen et al. (25) found that auricular pressure significantly reduced fasting insulin and C-peptide levels in patients, and Huang et al. (21) reported that auricular pressure significantly reduced the homeostasis model assessment-insulin resistance index (HOMA-IR) in patients. Fasting insulin and HOMA-IR are key indicators for assessing insulin resistance in patients with diabetes, while C-peptide levels reflect the secretory function of pancreatic β-cells. These findings indicate that auricular pressure may reduce blood glucose levels by increasing insulin sensitivity and regulating pancreatic β-cell function.

Meta-analysis indicated that auricular pressure significantly reduced BMI by 1.74 kg/m^2^ in type 2 diabetes patients. Given that obesity is one of the most important risk factors for type 2 diabetes (34), weight reduction support using auricular pressure may have powerful potential for the treatment of type 2 diabetes. In addition, auricular pressure also significantly reduced TC by 0.43 mmol/L, TG by 0.33 mmol/L, SBP by 14.75 mmHg, and DBP by 10.32 mmHg in patients with type 2 diabetes. Hyperlipidemia and hypertension are well known common risk factors for type 2 diabetes, and controlling blood lipid and blood pressure helps to reduce the risk of type 2 diabetes and delay disease progression (35). The lipid-regulating and blood pressure-lowering effects of auricular pressure may be effective in improving the prognosis of type 2 diabetes. Notably, the sensitivity analysis showed that significant difference in TG between the combined treatment and conventional treatment groups disappeared after removing the studies by Jian et al. (22) and Xu et al. (30). Out of the five included studies, the study by Jian et al. (22) had a significantly higher baseline TG level than the other studies, which may be an important reason for the difference in the results; we did not find obvious methodological or clinical heterogeneity between the study by Xu et al. (30) and the three other included studies. Given the lack of sufficient clinical evidence, we hope that more high-quality clinical trials will be conducted in the future to explore the effects of auricular pressure on blood lipid levels in patients with type 2 diabetes.

In addition to these outcome indicators, the included studies reported other benefits of auricular pressure. Shen et al. (25) found that auricular pressure significantly reduced Hamilton Anxiety Rating Scale and Hamilton Depression Rating Scale scores in patients with type 2 diabetes; these scales are commonly used to assess anxiety and depression. Previous studies have shown that anxiety and depression are common complications in patients with type 2 diabetes and are important factors affecting their quality of life (36). Depression and anxiety are also potential independent risk factors for type 2 diabetes (37, 38). This suggests that auricular pressure reduces the risk of comorbid anxiety and depression as well as the effects of anxiety and depression in patients with type 2 diabetes. In addition, Yang et al. (31) and Zheng et al. (18) found that auricular pressure effectively reduces the Pittsburgh Sleep Quality Index in patients with type 2 diabetes; the Pittsburgh Sleep Quality Index is commonly used to assess sleep quality, with higher scores indicating poorer sleep. Previous studies have shown that sleep disorders are associated with an increased risk of diabetic complications, and treating sleep disorders can help prevent the progression of diabetes (39). This finding implies that auricular pressure may further reduce the risk of complications in patients with type 2 diabetes by improving their sleep quality. Overall, auricular pressure can potentially reduce mood and sleep disorders in addition to lowering blood glucose, blood pressure, and blood lipids, helping to improve the prognosis of type 2 diabetes.

Subgroup analysis showed that auricular pressure treatment frequency of either less than or equal to once every 3 days and more than once every 3 days both significantly reduced FBG levels, suggesting that its hypoglycemic effect was not limited by treatment frequency. Both ≤ and > 30 days of auricular pressure significantly reduced FBG, suggesting that both short-term and long-term treatment have a hypoglycemic effect. Both ≤ and > 5 acupoints significantly reduced FBG, suggesting that the number of acupoints within the normal range did not limit the hypoglycemic effect of auricular pressure. This evidence suggests that the benefits of auricular pressure are not limited by treatment frequency, treatment duration, or the number of acupoints, implying that it has good variability in frequency, duration, and acupoint number that can allow clinicians to flexibly adjust the treatment plan according to the patient’s tolerance level.

### 4.3 Mechanism analysis

Little literature exists on the mechanisms of auricular pressure in type 2 diabetes. Previous studies have discussed the effects of auricular pressure on increasing insulin sensitivity and promoting insulin secretion. Wang et al. (40) found that auricular pressure directly stimulated the vagus nerve branch of the auricle and adjusted the balance of the autonomic nervous system, promoting insulin sensitivity and secretion. Liu et al. (41) reported that auricular pressure stimulates the thalamic sympathetic-adrenal and vagus insulin pathways by stimulating glucose- and insulin-sensitive neurons of the nucleus tractus solitarius, promoting insulin secretion from pancreatic beta cells.

### 4.4 Safety evaluation

Our meta-analysis showed that auricular pressure did not increase the incidence of additional adverse events, indicating that it is a relatively safe non-drug treatment. Despite this, clinicians must remain vigilant and prevent potential adverse events such as foreign body sensation, pain, infection, and allergy (42). Certain patients can experience a foreign body sensation, slight pain, or even bleeding when pressing beans on the auricle; this is typically caused by excessive or improper stimulation. Operators must adjust the stimulation intensity and method when performing auricular pressure to ensure patient comfort; when these conditions occur, stopping stimulation immediately will provide relief. Improper disinfection when applying auricular pressure or local skin damage caused by long-term pressure can lead to an infection. Standardized auricle disinfection is key to reducing the occurrence of infections. When an infection occurs, the ear patch should be removed immediately, and the auricle should be disinfected; topical or oral antibacterial drugs should be administered if necessary. Some patients may also experience local redness, swelling, itching, and other symptoms due to allergies to Wangbuliuxing seeds or tape. As this allergic reaction is most commonly caused by tape, replacing the tape with a desensitizing material can help reduce its occurrence. When an allergic reaction occurs, the ear patch should be torn off immediately, and the local skin should be washed with water or saline; antihistamines should be administered if necessary. Given that these potential adverse events are closely related to procedure operation, two suggestions could help to reduce the adverse events caused by operational factors and ensure the safety of auricular pressure; the first is to provide strict and standardized professional training to nursing staff performing auricular pressure measurements, while the second is to provide correct auricular pressure guidance to patients.

Auricular pressure is a form of acupuncture that is safe and reliable. Compared with oral drugs, auricular pressure does not involve hepatic metabolism and renal excretion, reducing the metabolic burden on the liver and kidneys. As a type of acupoint stimulation therapy, auricular pressure exerts a hypoglycemic effect through a neuroendocrine mechanism, which has fewer side effects than oral drugs that act on the whole body (43). In addition, auricular pressure is a relatively noninvasive form of stimulation compared to traditional needling or auricular acupuncture, as it does not involve piercing needles or other tools into the skin (44). A related systematic evaluation noted that the adverse events of auricular pressure were mainly foreign body sensation, pain, and local allergic reactions, and most were mild, short-term, and well-tolerated (42); the incidence of infection was very low.

### 4.5 Scheme analysis

**Figure 10:**
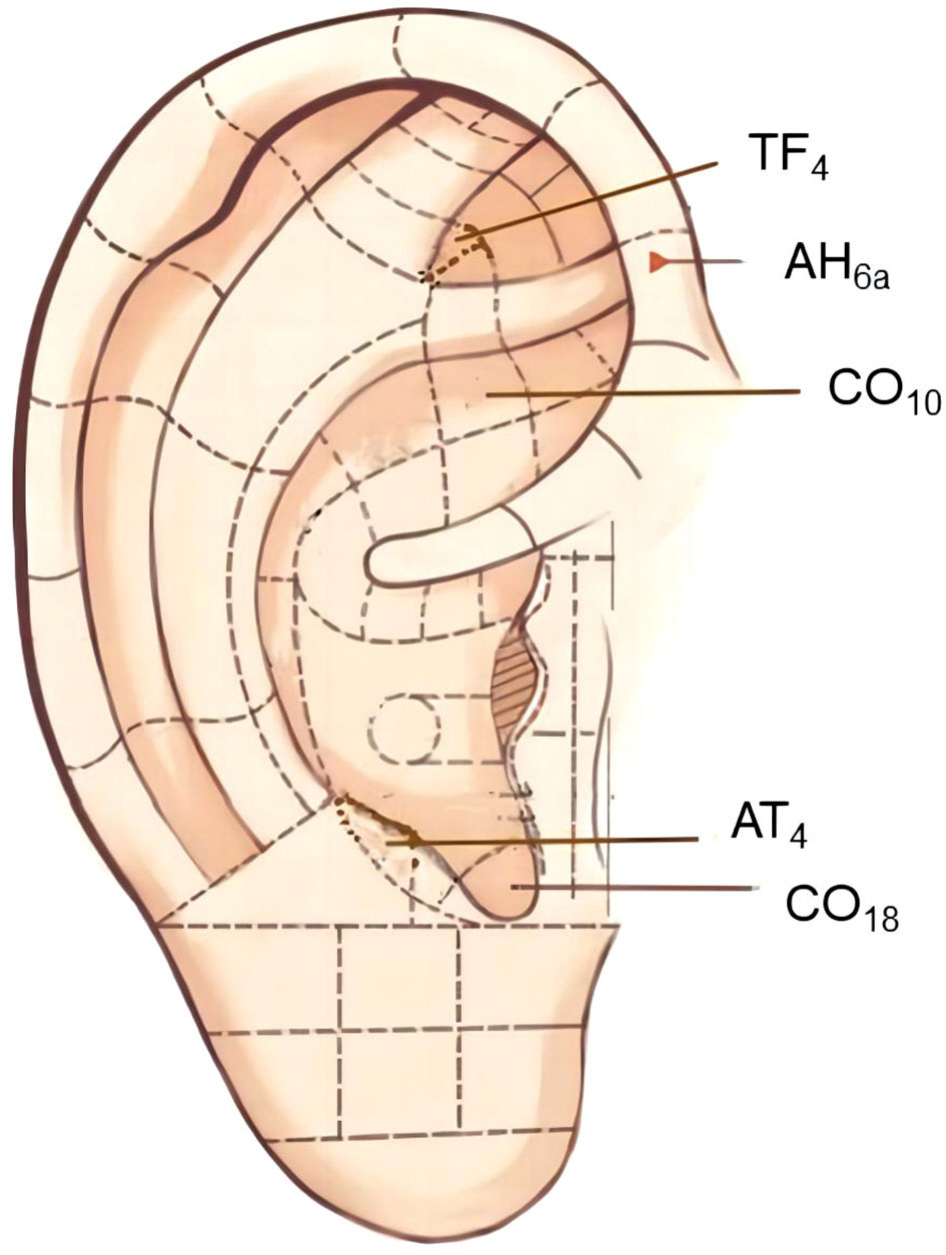
Localization of core acupoints.

Data mining revealed that AH_6a_, TF_4_, AT_4_, CO_18_, and CO_10_ are the core acupoints for auricular pressure treatment of type 2 diabetes; Auricular Therapy describes their localization and action (45). CO_18_ has a regulatory role in overall endocrine and immune functions, serving as a pivotal acupoint for rectifying endocrine dysfunction. AT_4_ is an important acupoint involved in the regulation of endocrine metabolism and digestive functions. CO_10_ serves as a core acupoint for health preservation, influencing the nervous and endocrine metabolic systems. AH_6a_ regulates autonomic nervous system function and vascular dilation. TF_4_ plays a role in tranquilizing the mind and regulating cardiovascular function. Together, they form a recommended plan for the treatment of diabetes using auricular pressure. The positioning of the auricular acupoints is shown in **Figure 10**.

Auricular pressure operations involved localization, disinfection, application of pressure pellets, daily pressure, and replacement of ear patches. The positions of AH_6a_, TF_4_, AT_4_, CO_18_, and CO_10_ should first be determined on the basis of an auricular acupoint chart and marked accordingly. Routine disinfection of the surface of acupoints should be performed with complex iodine. Ear patches should be applied to the aforementioned acupoints on one ear using clean tweezers. The patient should then gently press the ear patch with their hand until they experience sensations of soreness, numbness, distension, or warmth at the site; patients should be instructed to press each acupoint for 1 min at a time, 3 times a day. Ear patches should be replaced every 3–7 days; the ear patches should be gently removed, followed by wiping the ear with warm water to avoid allergic reactions caused by the adhesive tape residue.

### 4.6 Limitations and prospects

This research has some limitations. The included literature did not mention whether blinding of participants and personnel was used, which increases the potential risk of implementation bias. Sensitivity analysis showed that the combined results of TG were not robust, meaning that further verification from similar clinical trials is needed. Due to an insufficient study base, we were also unable to conduct a meta-analysis of effects on insulin, C-peptide, and HOMA-IR; because of this, the impact of auricular pressure on insulin secretion and insulin resistance remains unclear. Since the subjects included in the original studies were all Chinese, this study also may be unable to explain the role of auricular pressure in other ethnicities. Additionally, the auricular pressure scheme recommended in this study was obtained through data mining and required further validation in clinical trials.

In future research, experimental centers in other countries should be established to explore the effects of auricular pressure on patients of different races with type 2 diabetes. Additionally, insulin, C-peptide, and HOMA-IR should be added as outcome indicators to explore the impact of auricular pressure on these indicators in similar clinical trials. Additionally, the auricular pressure scheme recommended in this study should be tested in multicenter clinical trials to verify its efficacy and safety in the treatment of type 2 diabetes.

## 5 Conclusion

Auricular pressure safely improves blood glucose, lipids, blood pressure, and BMI in patients with type 2 diabetes compared to conventional treatment alone. The auricular pressure protocol developed through data mining that is proposed in this study, consisting of AH_6a_, TF_4_, AT_4_, CO_18_, and CO_10_, holds promise as a complementary treatment for type 2 diabetes.

## Data Availability

The original contributions presented in the study are included in the article/supplementary material. Further inquiries can be directed to the corresponding author.
